# Hyperthermal velocity distributions of recombinatively-desorbing oxygen from Ag(111)

**DOI:** 10.3389/fchem.2023.1248456

**Published:** 2023-08-02

**Authors:** Arved C. Dorst, Rasika E. A. Dissanayake, Daniel Schauermann, Sofie Knies, Alec M. Wodtke, Daniel R. Killelea, Tim Schäfer

**Affiliations:** ^1^ Institute of Physical Chemistry, University of Göttingen, Göttingen, Germany; ^2^ Max-Planck Institute for Multidisciplinary Sciences, Göttingen, Germany; ^3^ Faculty of Biology, Chemistry and Geosciences and Bavarian Center for Battery Technology, Bayreuth, Germany; ^4^ Department of Chemistry and Biochemistry, Loyola University Chicago, Chicago, IL, United States

**Keywords:** oxygen, silver, ion imaging, TPD, velocity-resolved, molecular beams, angular distribution, energy distribution

## Abstract

This study presents velocity-resolved desorption experiments of recombinatively-desorbing oxygen from Ag (111). We combine molecular beam techniques, ion imaging, and temperature-programmed desorption to obtain translational energy distributions of desorbing O_2_. Molecular beams of NO_2_ are used to prepare a *p* (4 × 4)-O adlayer on the silver crystal. The translational energy distributions of O_2_ are shifted towards hyperthermal energies indicating desorption from an intermediate activated molecular chemisorption state.

## 1 Introduction

Silver surfaces play important roles in large scale industrial heterogeneous catalytic processes such as partial oxidation of methanol to formaldehyde and ethylene to ethylene oxide (Serafin et al., 1998; Qian et al., 2003). Because of the tremendous scale of these applications, seemingly modest improvements in the reaction process may lead to big economical and ecological improvements. Therefore, this system has attracted significant attention over the years and numerous studies have focused on the microscopic details of oxidized silver surfaces. Oxygen induced reconstructions of silver surfaces have been thoroughly investigated using high-precision ultra-high vacuum (UHV) surface science techniques in combination with theoretical approaches (Bao et al., 1996; Michaelides et al., 2005; Schnadt et al., 2006; Greeley and Mavrikakis, 2007; Reichelt et al., 2007; Rocha et al., 2012; Martin et al., 2014; Jones et al., 2015a; Jones et al., 2015b). Ag (111) exhibits a variety of different reconstructed surfaces with similar stability which have been studied and discussed for many years. A detailed review about the history of considered oxygen structures on Ag (111) is given by Michaelides et al. (2005).

Experimentally, the oxidation of Ag (111) under UHV conditions with molecular oxygen is difficult due to the low sticking probability (ca. 1 × 10^−6^) of O_2_ (Campbell, 1985; Kleyn et al., 1996). In early UHV studies, silver surfaces were therefore oxidized under comparatively high O_2_ pressures before characterization under UHV conditions (Campbell, 1985). The use of more aggressive oxidants circumvents this issue; in particular, atomic oxygen (Bukhtiyarov et al., 2003; Böcklein et al., 2013; Derouin et al., 2015) or NO_2_ (Bare et al., 1995; Huang and White, 2003) allow for silver surface oxidation under UHV compatible conditions. When using NO_2_ as oxidant, the temperature range at which clean oxidized surfaces are produced is restricted between ca. 490 K and 520 K since at lower temperatures NO_2_ adsorbs molecularly and at elevated temperatures, O_2_ starts desorbing (Huang and White, 2003). When oxidizing at these temperatures, the reconstructed surface is indistinguishable from surfaces oxidized with molecular oxygen and consists mainly out of *p* (4 × 4)-O domains (Carlisle et al., 2000a; Carlisle et al., 2000b). In contrast, oxidizing with atomic oxygen is possible at lower temperatures. It typically results in slightly different surface phases and forms subsurface oxygen below 510 K, (Derouin et al., 2015).

The large number of reconstructed oxidized Ag (111) surfaces observed in experiments has motivated theory groups to develop models describing surface stability based on first principles theory (Michaelides et al., 2003; Li et al., 2003a,b; Michaelides et al., 2005). Using *ab initio* thermodynamics and first principles simulations, theory is able to provide (*T*, *p*) phase diagrams describing stable oxidized surface phases from UHV to high pressure conditions present at real world catalysts (Reuter, 2016). By comparison with experimental results, microscopic details of the oxidized surface structure can be elucidated.

Additional theoretical work has focused on the dynamics of the O_2_ dissociation process on Ag (111). These studies do not aim for clarifying the geometry of reconstructed surfaces but provide theoretical data on the atomic scale mechanism of the oxidation process itself (Xu et al., 2005; Kunisada et al., 2011; Kunisada and Sakaguchi, 2014). Kunisada and Sakaguchi investigated quantum dynamics of O_2_/Ag (111) dissociative adsorption propagating on a six-dimensional potential energy surface (PES) obtained from density-functional theory (DFT) (Kunisada and Sakaguchi, 2014). From the PES, they identify the lowest barrier near a top site with a height of 1.37 eV. Coupled-channel calculations trajectories based on this PES provide dissociation probabilities for O_2_ as function of the incident translational and vibrational energy. Interestingly, dissociation occurs even with translational energies slightly below the activation barrier height, which the authors explain by O_2_ tunneling effects. The computations also show a significant dissociation enhancement by increasing the incident vibrational energy caused by a late barrier in the reaction pathway. In another theoretical study based on a neural network interpolated PES, Goikoetxea et al. investigated electronically non-adiabatic effects during the dissociative adsorption of O_2_ at Ag (111) (Goikoetxea et al., 2012). They also identified a large energy barrier for dissociation above 1 eV close to the surface. As non-adiabatic effects affect sticking probabilities at elevated distances to the surface and are expected to be smaller than the adiabatic energy barrier, their influence on the sticking probability is negligible.

Such theoretical work provides excellent data for comparison with surface dynamics experiments under well-controlled UHV conditions. A classical experimental approach probes the entrance channel of the reaction pathway by employing pulsed molecular beams of reactants to initiate the surface reaction (Barker and Auerbach, 1984; Kleyn et al., 1996; Sitz, 2002; Kleyn, 2003; Golibrzuch et al., 2015; Chadwick and Beck, 2016; Vattuone and Okada, 2020; Shen et al., 2022). Seeding reactants in different carrier gases allows for modification of the incident translational energy. Incident vibrational energy can be altered by thermal or laser excitation. Surface reactivity as function of varied incident parameters is probed using, for instance, Meitner-Auger electron spectroscopy (MAES) or temperature -programmed desorption (TPD) for coverage determination after exposing the surface for a selected time to a molecular beam.

Surface reaction dynamics experiments on the exit channel probe degrees of freedom of the desorbing reaction products using quantum state-resolved detection methods in combination with translational energy dependent measurements (Comsa and David, 1982; Michelsen and Auerbach, 1991; Michelsen et al., 1992; Shuai et al., 2017; Kaufmann et al., 2018; Dorst et al., 2022). From these studies, translational, rotational, and vibrational state distributions of products can be deduced. Eventually, concepts of detailed balance allow to model these distributions and to defer quantitative heights of reaction barriers.

Recently, we used this approach to investigate the recombinative desorption of oxygen from Rh (111) (Dorst et al., 2022). O_2_ was detected using a velocity map imaging (VMI) setup after non-resonant ionization with a femtosecond pulse of 800 nm. The desorption process was initiated by linearly heating the sample in a TPD type approach. We identified hyperthermal velocity distributions for oxygen molecules desorbing from surface sites as well as for oxygen molecules originating from subsurface, indicating a common intermediate desorption state.

In this paper, we present angular distributions and translational energy distribution of recombinatively-desorbing O_2_ from Ag (111). We prepare *p* (4 × 4)-O Ag (111) by dosing the surface with a molecular beam of NO_2_ seeded in rare gases at a surface temperature of 510 K. Angular distributions are narrow and hyperthermal translational energy distributions indicate an activated desorption process.

## 2 Experimental

The experimental setup has been previously described in detail (Westphal et al., 2020). Briefly, experiments were conducted under ultra-high vacuum (UHV) conditions at a base pressure of <5 × 10^−10^ mbar. We dose the surface with a pulsed supersonic molecular beam of 10% NO_2_ (AirLiquide, 99.5%) seeded in He (AlphaGaz, ≥99.999%) using a home-built pulsed solenoid nozzle (Park et al., 2016). During exposure, we maintain UHV conditions by differential pumping techniques.

For surface cleaning, the UHV apparatus is equipped with an ion gun (Staib Instruments IG-5-C), with which the surface is Ar^+^-sputtered (2.00 kV, 2.0 × 10^−7^ mbar Ar) for multiple cycles. After annealing (700 K, 30 min), the surface cleanliness is checked by Meitner-Auger electron spectroscopy (OCI BDL 450) and low-energy electron diffraction (LEED) spectroscopy (OCI BDL 450). The Ag (111) crystal (MaTecK, 99.99%, *∅* 10 mm, 2 mm thickness) is mounted on a home-built sample holder and is resistively heated by Ta filaments; temperatures are monitored by a K-type thermocouple. With liquid nitrogen cooling, the accessible temperatures range from 100 K to 1,235 K. We use a home-written LabVIEW™ program for data acquisition and control of experimental parameters.

**FIGURE 1:**
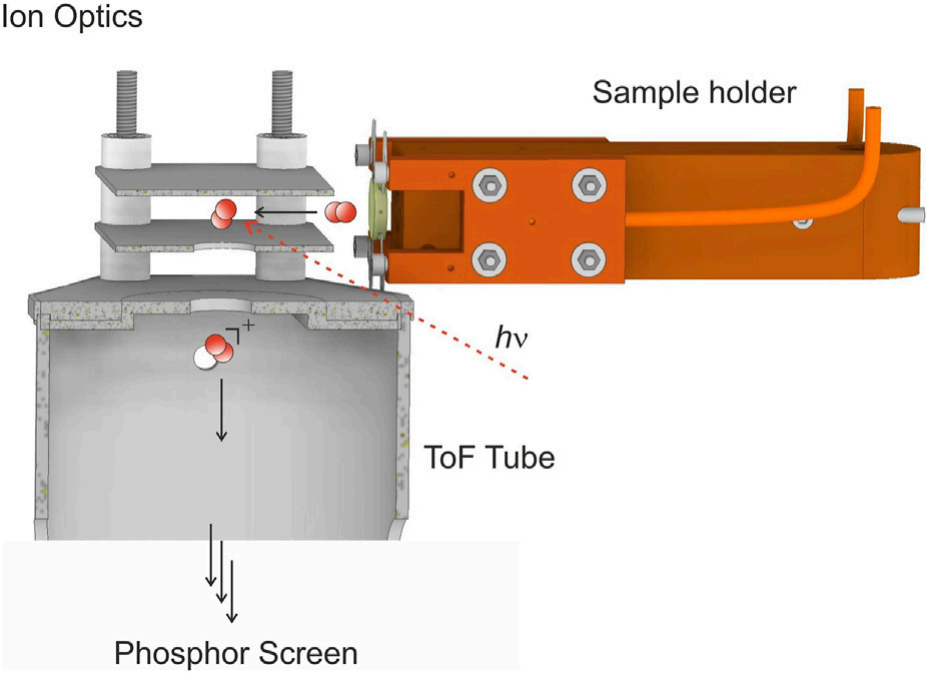
The surface temperature is linearly ramped by resistive heating. Recombinatively-desorbing molecules are ionized using non-resonant multi-photon ionization and coupled into the time-of-flight (ToF) tube by the ion optics of the velocity map imaging detector. Ions are detected on a phosphor screen at the end of the ToF tube (not shown). Velocities of desorbing molecules are deduced from the position at which the ions hit the phosphor screen.

Velocity distributions of surface desorption products are obtained by combining velocity map imaging (VMI) and temperature-programmed desorption (TPD) experiments: we linearly heat up the surface while simultaneously detecting velocity map images of desorbing molecules. For that, the beam of a regenerativly amplified femtosecond laser (Spectra-Physics, Solstice Ace, <35 fs, 800 nm, 1 kHz) is focused by an optical lens (*f* = 300 mm) such that molecules are non-resonantly ionized after desorption. The ions are detected by an imaging setup which follows the design by Eppink and Parker (Eppink and Parker, 1997). Micro-channel plates (MCPs, Topag, MCP 56–15) are used for signal amplification and ions are imaged using a CMOS camera (Basler ace acA 1,920–155 μm, 1,920 px × 1,200 px) recording the images from a phosphor screen (Proxivision P43). Figure 1 shows the ionization region of the experimental setup.

Before each TPD experiment, the sample is exposed to the molecular beam at a defined dosage temperature. Afterwards, the surface is linearly heated at 4 K s^−1^ in a TPD experiment while we record images at 1 kHz laser repetition rate.

## 3 Results and discussion

**FIGURE 2:**
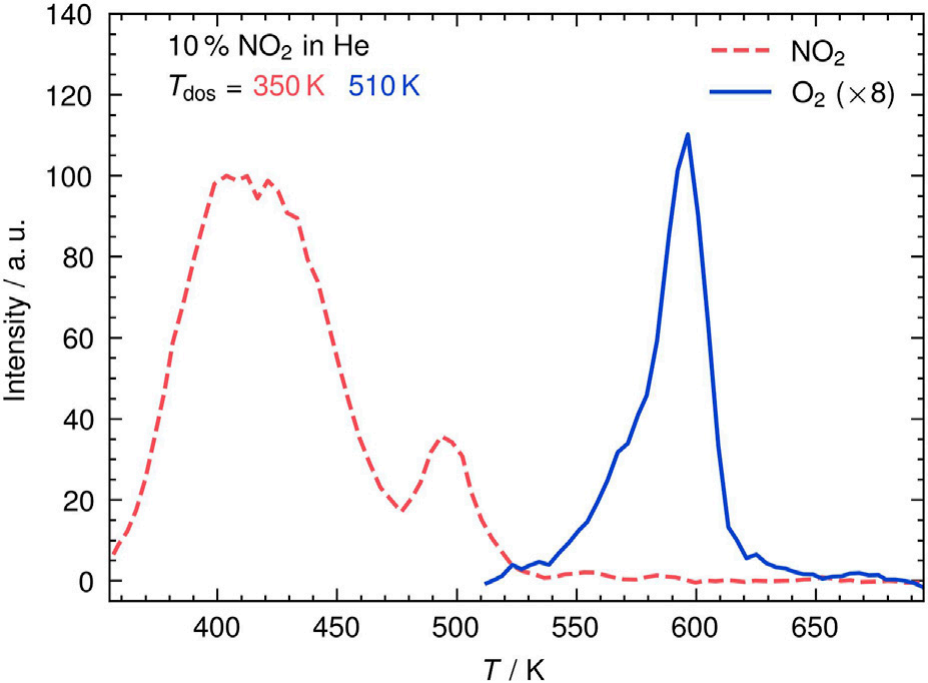
VMI-TPD spectrum of desorbing NO_2_ (dashed) and recombinatively-desorbing O_2_ (solid) after identical NO_2_ exposure of an Ag (111) surface. The integrated flux density is plotted as a function of the temperature *T*; *T*_dos_ is the dosage temperature.

For investigating velocity-resolved desorption of recombinatively-desorbing oxygen, we first create a complete monolayer of a *p* (4 × 4)-O phase on Ag (111) by dosing it with NO_2_ from a molecular beam at *T*_surf_ = 350 K or 510 K for 2 min at a nozzle frequency of 200 Hz. We check the degree of oxidation by LEED and TPD (see Supplementary Material). Depending on the surface temperature during exposure, either nitrate (NO_3_) or pure oxygen layers may form (Alemozafar and Madix, 2005). In Figure 2, we show TPD spectra of NO_2_ and O_2_ recorded with the VMI setup displayed in Figure 1. We record the total signal at the phosphor screen of the molecular mass of the parent ion by gating the phosphor screen to the respective time-of-flight. The VMI-TPD spectrum of NO_2_ (dashed curve) shows two different desorption features after dosing at 350 K: first, a broad desorption peak ranging up to 470 K followed by a less broad, lower intensity desorption between 480 K and 510 K. These features are attributed to NO_3_ decomposition into NO_2_(g) and O from two different states (Alemozafar and Madix, 2005). In contrast, O_2_ (solid curve) desorbs at significantly higher temperatures at *T*_surf_ of ≈590 K (Huang and White, 2003).

**FIGURE 3:**
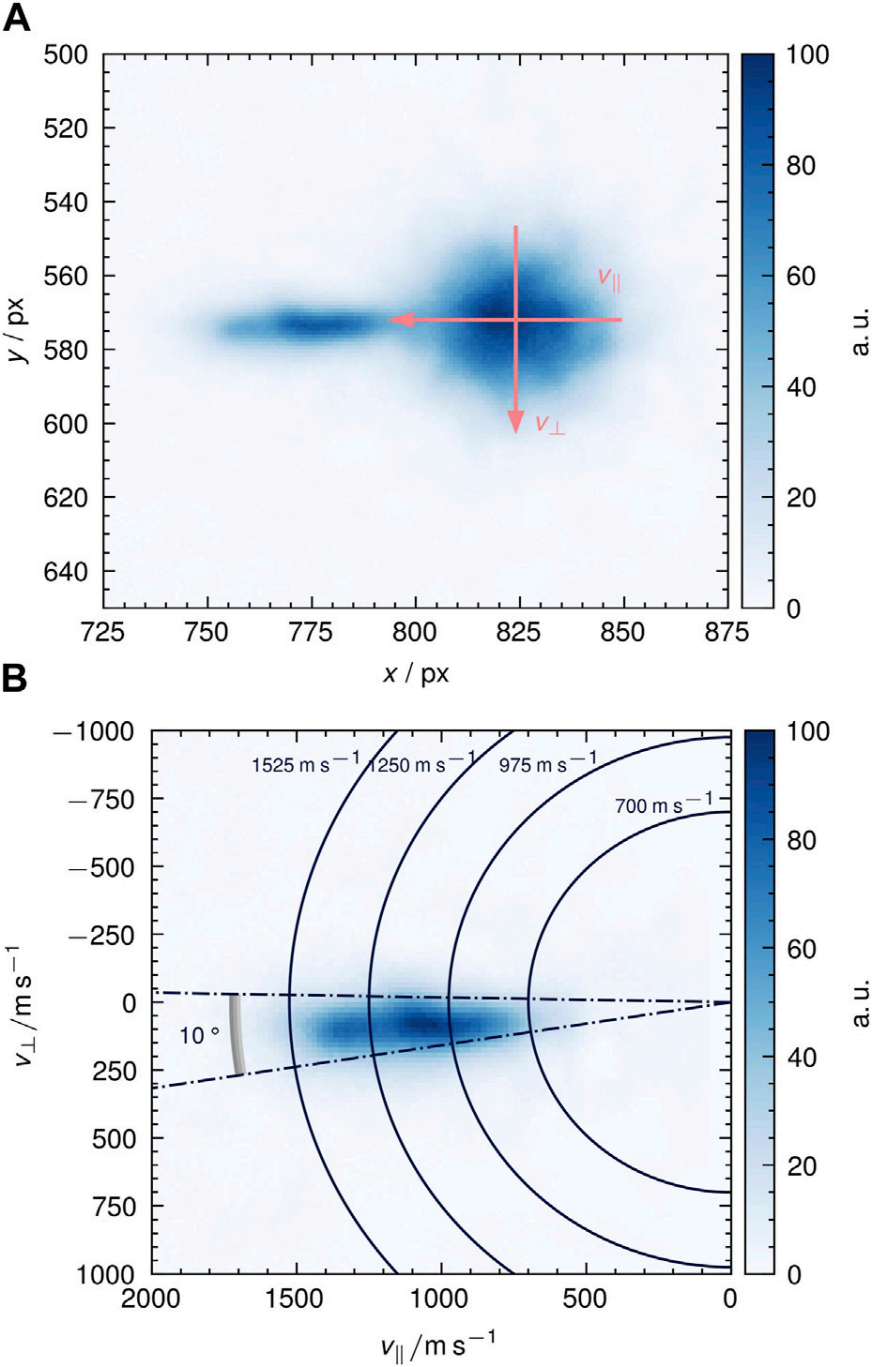
Velocity-map images of recombinatively-desorbing O_2_ from Ag (111) around 600 K. *v*_⊥_ and *v*_‖_ are defined relative to the surface normal. **(A)** Raw velocity-map image with indicated thermal background. We use the thermal background for defining the point of zero velocities and to calibrate the detector. For that, we fit the room temperature thermal background with a 1-D Maxwell-Boltzmann distribution. The width of the distribution provides the calibration and the center provides zero velocity. One can clearly distinguish the hyperthermal velocities of surface desorbing oxygen and the background. **(B)** Velocity-mapped image after thermal background subtraction, *v* calibration, and density-to-flux conversion.

**FIGURE 4:**
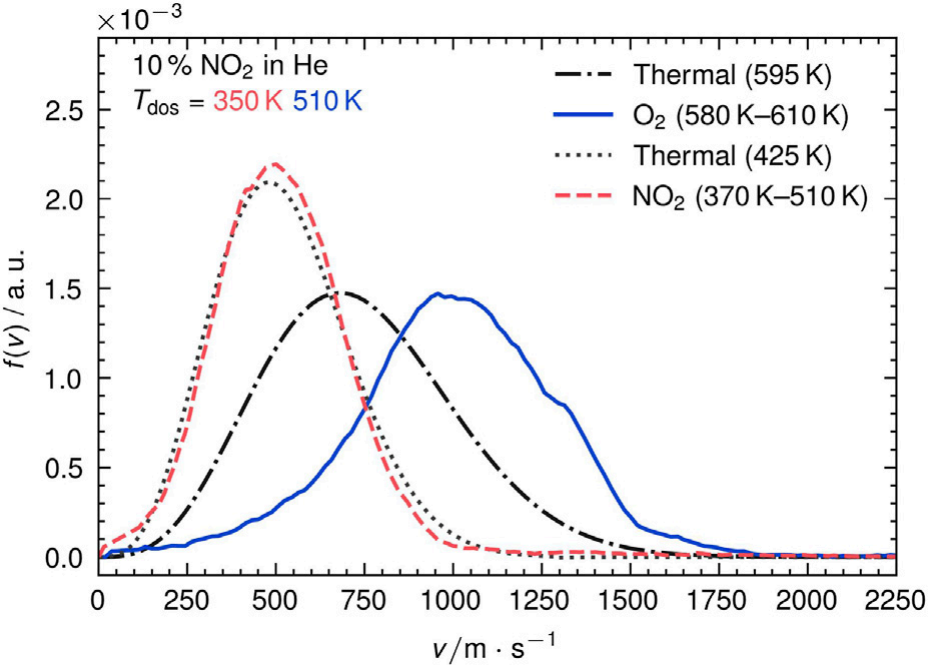
Plot of the experimental velocity distributions *f*(*v*) for NO_2_ and O_2_ against the velocity *v*. For comparison, thermal distribution at the desorption peak’s temperature are shown. All curves are normalized to an integral of one.

We use the VMI setup to determine velocity distributions for both, desorbing NO_2_ and recombinatively-desorbing O_2_. Figure 3A shows the raw image of O_2_ desorption from Ag (111) around 590 K. We obtain the image by averaging all images that we record during a desorption peak in a TPD run. The raw image clearly displays the residual thermal gas background in the UHV chamber as circular spot as indicated in Figure 3A. From the background we deduce the point of zero velocity. We further calibrate the detector by fitting a one-dimensional Maxwell-Boltzmann distribution to the thermal background. Figure 3B shows the calibrated figure after subtraction of the thermal background and density-to-flux conversion (Harding et al., 2017). The velocity-mapped image shows a directed desorption feature with hyperthermal velocities between 500 m s^−1^ and 1,500 m s^−1^. The angular tilt is due to a slightly tilted suspension of the crystal in the sample holder. From such images, we deduce velocity distributions by iterative integration over velocity increments within 10◦-broad angular slices as shown in Figure 3B. In Figure 4, we show the results for the NO_2_ peak at 425 K and the O_2_ peak at 595 K. All curve integrals are normalized to unity. For comparison, thermal flux-weighted Maxwell-Boltzmann distributions of the shape

$$f\left(v,T_{\text{surf}}\right)\propto v^{3}\cdot \exp\left(-\frac{M\cdot v^{2}}{R\cdot T_{\text{surf}}}\right)$$ (1)

are plotted. *M* denotes the molar mass of the compounds and *R* is the universal gas constant. We use the signal-weighted temperature ⟨*T*⟩ of 425 K for NO_2_ and 595 K for O_2_ for *T*_surf_.

From the figure it is obvious that O_2_ desorbs with hyperthermal velocities indicated by a shift of the curve’s maximum by more than 300 m s^−1^ compared to a flux-weighted thermal velocity distribution. In contrast, NO_2_ desorption is clearly thermal as it can be well-reproduced by a flux-weighted Maxwell-Boltzmann distribution of the surface temperature. We did not observe any significant difference between the two desorption features of NO_3_ decomposition (see Supplementary Material). Table 1 lists the characteristic properties of the shown velocity distributions.

**TABLE 1:** Mean experimental velocities ⟨*v*⟩, energies ⟨*E*⟩, and the corresponding mean thermal velocities ⟨*v*_th_⟩ for a desorption temperature ⟨*T*⟩ of desorbing NO_2_ and recombinatively-desorbing O_2_ from Ag (111).

Compound	⟨*T*⟩/K	⟨*v*⟩/m s^−1^	⟨*v*_th_⟩/m s^−1^	⟨*E*⟩/eV
NO_2_	425	530	520	0.0731
O_2_	595	1,010	795	0.186

**FIGURE 5:**
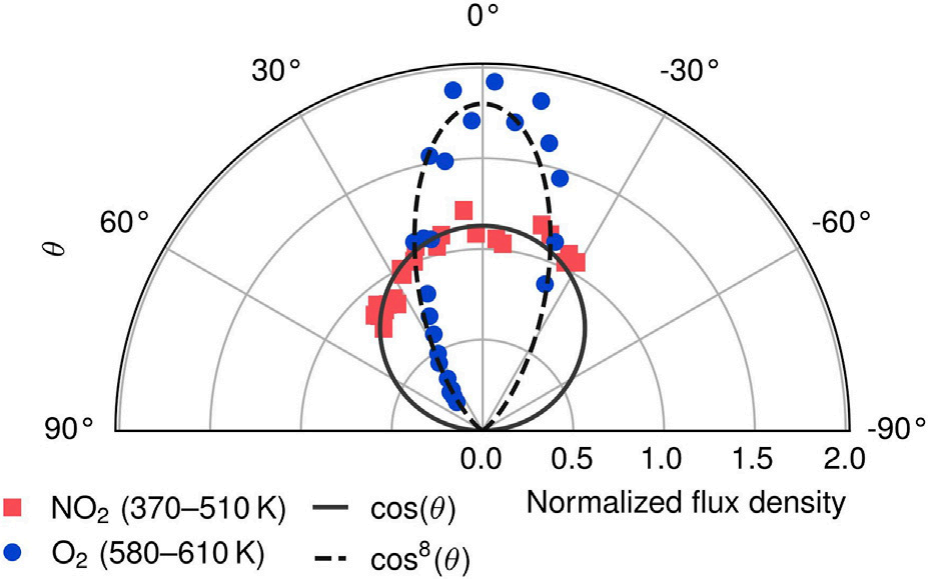
Polar plot of angular resolved flux for distinct TPD peaks for NO_2_ (squares) and O_2_ (circles) desorption from Ag (111) after NO_2_ exposure. Thermal cos(*θ*) (solid) and non-thermal cos^8^(*θ*) (dashed) distributions are shown for comparison. The integrals are normalized to one.

Additionally, we record angular distributions of NO_2_ and O_2_ desorption from Ag (111). For that, we move the surface parellel to the detector such that only molecules from certain desorption angles are detected as described previously (Dorst et al., 2022). As VMI provides the direction of velocities in the detector plane, desorption angles are directly obtained from the ion images. Figure 5 shows a polar plot of the angular resolved flux for both, NO_2_ (squares) and O_2_ (circles) desorption. For comparison, a cos(*θ*)-distribution is shown, which would be expected for thermal desorption. We observe a narrow cos^8^(*θ*)-angular distribution for O_2_ desorption, whereas NO_2_ desorption resembles the cos(*θ*)-distribution indicative of a thermalized (or equilibrium) desorption process.

From the presented desorption dynamics, details of the underlying potential energy surface (PES) are obtained. The narrow angular distribution of O_2_ desorption indicates an activated desorption process. Molecules have to overcome a barrier, on which they get accelerated into the gas phase. This is known to result in very peaked angular distributions (Comsa and David, 1985). In contrast, the release of NO_2_ after surface nitrate decomposition appears with a broad angular distribution, indicating a non-activated thermalized desorption process.

**FIGURE 6:**
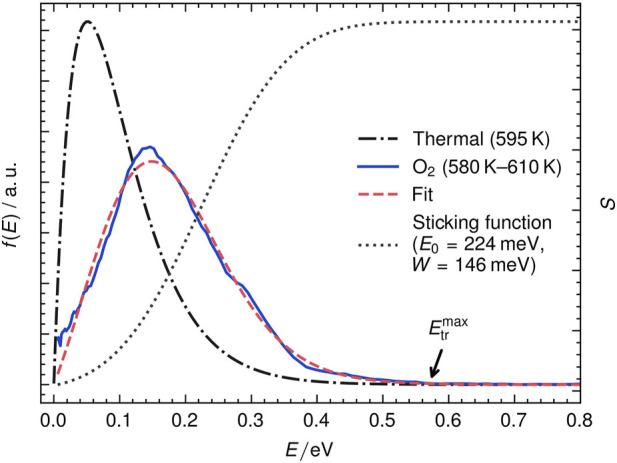
The translational energy distribution of recombinatively-desorbing O_2_ (solid line) from Ag (111) can be modeled with the product (dashed) of a thermal energy distribution (dash-dotted) and a sticking function *S* (dotted). Highlighted is the maximum observed translational energy $E_{\text{tr}}^{\text{max}}$ . *E*_0_ is the energy barrier and *W* the width parameter in Eq. 3.

Also, velocities of desorbing molecules provide valuable information about the underlying PES. Figure 6 shows the translational energy distribution of recombinatively-desorbing O_2_ from Ag (111). The distributions are obtained from the velocity distributions displayed in Figure 4. The O_2_ distribution is clearly shifted towards higher energies; NO_2_ resembles a thermal distribution.

The shift of the hyperthermal O_2_ energy distribution can be used to quantify energy-dependent sticking probabilities using the concepts of detailed balance (White and Beuhler, 2004). In an activated adsorption process, an energy barrier in the adsorption trajectory suppresses sticking at low incident translational energies. As a consequence, molecules with low translational energy are missing in translational energy distributions of desorption (Comsa and David, 1982; Michelsen and Auerbach, 1991; Shuai et al., 2017; Kaufmann et al., 2018). Following the principles of detailed balance, we can fit the hyperthermal distribution in Figure 6 using the product of a flux-weighted thermal energy distribution and a sticking function (see Eq. 2).

$$f\left(E_{\text{tr}},T_{\text{surf}}\right)=K\cdot E_{\text{tr}}\cdot \exp\left(-\frac{E_{\text{tr}}}{k_{\text{B}}\cdot T_{\text{surf}}}\right)\cdot S\left(E_{\text{tr}}\right)$$ (2)

Here, *K* is a constant factor, *E*_tr_ is the translational energy of desorbing molecules, *T*_surf_ is the surface temperature and $S\left(E_{\text{tr}}\right)$ is the sticking function. We apply an error function to describe sticking (see Eq. 3) as has been done in previous studies for activated adsorption processes and plot the sticking function in Figure 6 as dashed lines (Michelsen and Auerbach, 1991; Michelsen et al., 1992; Luntz, 2000).

$$S\left(E_{\text{tr}}\right)=\frac{1}{2}\left(1+erf\left(\frac{E_{\text{tr}}-E_{0}}{W}\right)\right)$$ (3)

*W* represents the width and *E*_0_ represents the inflection point. We fit our data with a *E*_0_ = 0.224 eV as shown in Figure 6. Not that the inflection point corresponds to the onset of adsorption and is related to the energy barrier height. This height is often strongly dependent on the adsorbate’s rotational and vibrational state. State-resolved permeation studies on energy distributions of the H_2_/Cu(111) system reveal for instance significant enhanced sticking probabilities for vibrationally excited molecules (Michelsen and Auerbach, 1991). However, in this work, we universally ionize desorption products without quantum state resolution. The translational energy distribution should therefore be considered as an averaged distribution of different states with unknown populations. We define also the maximum observable O_2_ translational energy $E_{\text{tr}}^{\text{max}}$ similar to the method developed by Fingerhut et al. (2021). In their work on formate decomposition on hydrogenated Pt (111), they identified $E_{\text{tr}}^{\text{max}}$ as lower limit of the energy barrier in the entrance channel.

We indicate this threshold as black arrow in Figure 6 at ca. 0.57 eV (≈1850 m s^−1^). For these fast molecules, we assume no internal energy and that the recoil against the surface from the transition state results only in minor Ag phonon excitation. This value should therefore be the lower limit to the real energy barrier height as we do not account for excitation of the solid.

Interestingly, these values are significantly lower than calculated sticking probabilities based on first principles theory (Goikoetxea et al., 2012; Kunisada and Sakaguchi, 2014). Kunisada and Sakaguchi calculate state-resolved sticking by performing quantum dynamics calculations of O_2_ dissociative desorption on Ag (111) on a before computed PES (Kunisada et al., 2011). Depending on the adsorption site, they predict the onset of adsorption between 1.2 eV and 2.1 eV O_2_ incident energy. They also calculate a significant influence of vibrational excitation on the dissociation probability by reducing the onset by ca. 30% when comparing O_2_ (*v* = 0) to O_2_ (*v* = 3). However, this reduction is still not sufficient to explain the discrepancy between the experimentally measured onset of this study of 0.57 eV and the minimum value of 0.8 eV (O_2_ (*v* = 3) for a bridge site) of the theoretical work. We therefore suspect that we do not map a direct dissociation trajectory but desorption from another intermediate surface state under the experimental conditions applied in this study.

In a systematic molecular beam surface scattering approach, Kleyn et al. studied the interaction of O_2_ molecular beams with Ag (111) at 150 K identifying several scattering pathways using ToF detection techniques (Raukema and Kleyn, 1995; Kleyn et al., 1996; Raukema et al., 1997). From the ToF of scattered O_2_, translational energy distributions are obtained, which can be attributed to different surface states prior to desorption. In general, for the O_2_/Ag (111) system, three adsorption states exist: a shallow physisorption well, a molecular chemisorption well, and a dissociative chemisorbed state (Campbell, 1985). At low incident translational energies, scattered oxygen exhibits low velocities, indicating desorption from the physisorbed state. At elevated incident energies, two significantly faster scattering channels are observed. The fastest channel depends on the incident energy indicating directly scattered O_2_. In contrast, the other fast channel is independent on the incident energy, so that the authors assign this pathway to transient trapping desorption from the molecular chemisorption potential energy well. Adsorption in this state is activated with a threshold mean energy of about 0.2 eV, and exhibits electron transfer from the surface to the adsorbate. The molecular chemisorption state can serve as precursor state for dissociative chemisorption (Kleyn et al., 1996). Recent calculations indicate an energy barrier of 0.8 eV between both states (Hinsch et al., 2021). The mean final energy of O_2_ molecules originating from the molecular chemisorption state recorded by Kleyn et al. is 0.14 eV (Kleyn et al., 1996). It is close to 0.19 eV, which we measured in this study. The lower translational energy could be caused by the significant colder surface temperature of 150 K. This is indication that the desorption state, which we observe in TPD experiments at 590 K, is identical to the intermediate molecular chemisorption state observed in molecular beam surface scattering experiments.

## 4 Conclusion

We performed velocity resolved surface desorption experiments of recombinatively desorbing O_2_ from Ag (111) by combining ion imaging techniques with temperature programmed desorption. Desorption occurs at 590 K, is clearly hyperthermal, and exhibits a narrow angular distribution indicating an activated desorption process. Velocity distributions are similar to previously reported distributions from molecular beam surface scattering experiments. For both studies, the energetics of desorbing molecules indicate desorption from an intermediate molecular chemisorption state. Recent theoretical papers calculate significantly higher barriers for oxygen sticking on Ag (111) than we deduce from the translational energy distribution (Kunisada and Sakaguchi, 2014). The here presented data will be a valuable experimental benchmark to refine theoretical models crucial for a better understanding of surface dynamics in metal oxidation processes.

## Data Availability

The raw data supporting the conclusion of this article will be made available by the authors, without undue reservation. It is available under GRO.data (https://data.goettingen-research-online.de/dataverse/hyperthermal_v_distr_desorbing_o2_ag111). Further inquiries can be directed to the corresponding author.
