# Supplementary material for: Hyperthermal velocity distributions of recombinatively-desorbing oxygen from Ag(111)
Source: Front Chem. 2023 Aug 2;11:1248456. doi: 10.3389/fchem.2023.1248456 (PMC10433164; doi:10.3389/fchem.2023.1248456)
Supplement: Supplementary file 1 [file DataSheet1.PDF]

Supplementary Material

1 SUPPLEMENTARY TABLES AND FIGURES

1.1 LEED images

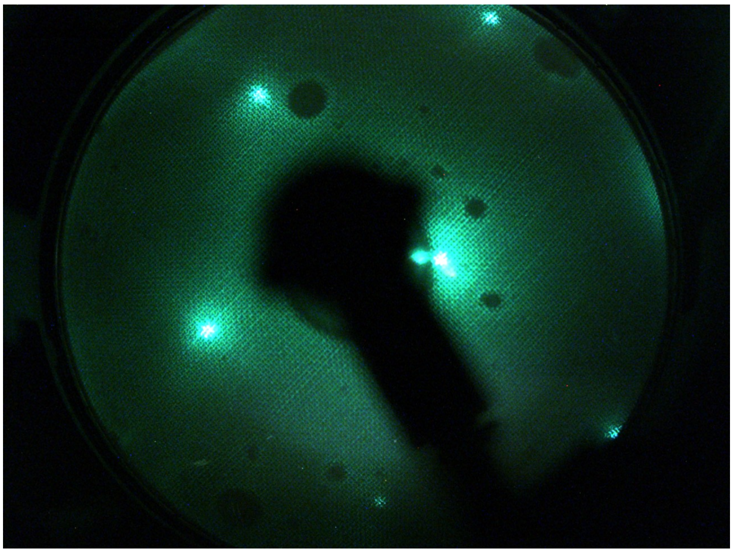

Figure S1. LEED image of the clean Ag(111) surface recorded at a beam energy of 50 eV.

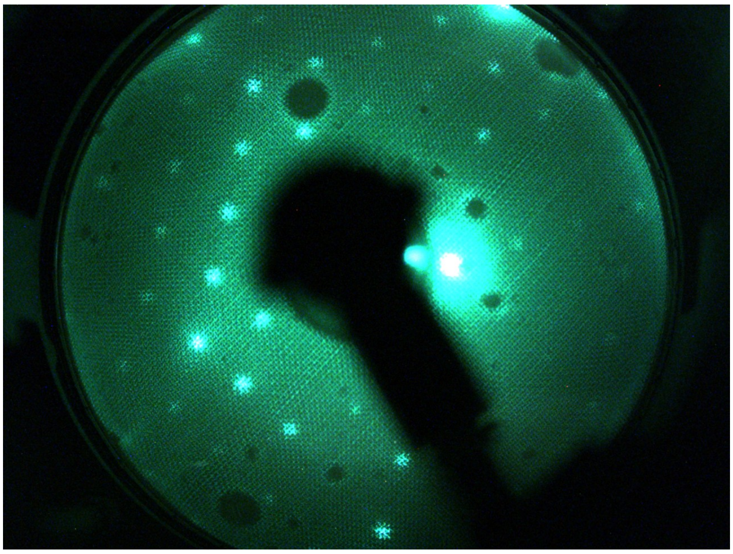

Figure S2. LEED image of the Ag(111) surface recorded at a beam energy of 50 eV after dosage of NO_2 at 510 K.

1.2 Velocity distributions of NO_2 desorption features

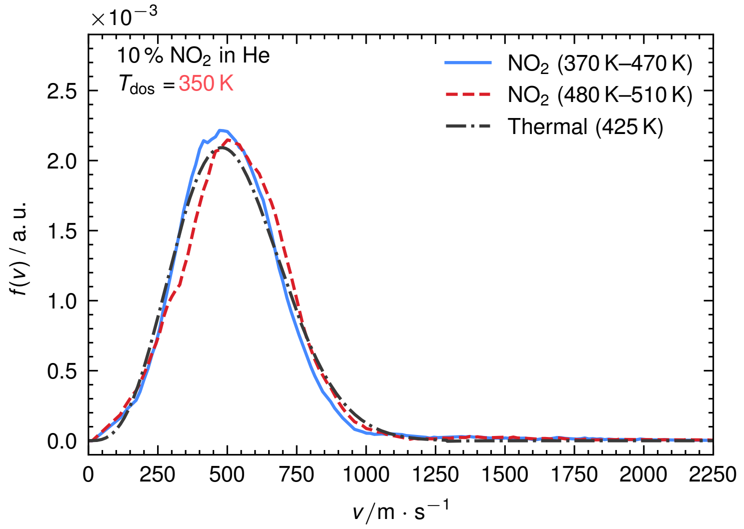

Figure S3. Velocity distributions $f(v)$ as a function of the velocity v for both NO_2 desorption features in Figure 4. Both distributions exhibit the same mean velocity within an uncertainty of below 3 %. A flux-weighted thermal Maxwell-Boltzmann distribution at 425 K is shown for comparison. All curve integrals are normalized to one.
